# Spring Tonics

**Published:** 1891-07

**Authors:** 


### SPRING TONICS.

Of the many positive antiseptic medicines that antidote the disease germs, one of the most simple and valuable, is sulphur; it is a powerful antiseptic and rapidly destroys the bacteria and diseased germs in the blood. This is the reason why sulphur is unrivaled in the treatment of diphtheria and kindred diseases. It is also very valuable in the treatment of chronic rheumatism, piles, boils, and eruptions of the skin. As a spring remedy, it should be combined with cream of tartar, as follows: ℞ Sulphur, 6 teaspoonfuls; cream of tartar, 3 teaspoonfuls. Mix.

One teaspoonful of the above powder mixed in molasses or syrup should be taken by an adult every morning for a week or ten days; then omit for a week, after which it should be repeated as before. Simultaneously with the administration of the above, we would suggest the administration of the second valuable remedy for this season, viz: muriate tincture of iron. This we would give in small doses, say five to eight drops in a wineglass of water after each meal, during the time the sulphur is being taken. Always take the iron through a straw or tube so it shall not come in contract with the teeth. After these remedies have been taken, give quinine twice a day, from one-half to two grain doses, according to whether you live in a malarial region or not. The proper administration of these old, well tried remedies will prove all sufficient in almost all cases where it is essential to give any medicine.

